# Observed vulnerability of Filchner-Ronne Ice Shelf to wind-driven inflow of warm deep water

**DOI:** 10.1038/ncomms12300

**Published:** 2016-08-02

**Authors:** E. Darelius, I. Fer, K. W. Nicholls

**Affiliations:** 1 Geophysical Insitute, University of Bergen and the Bjerknes Centre for Climate Research, Allég. 70, 5007 Bergen, Norway; 2 UNI Research Climate, Nygårdsgaten 112–114, 5008 Bergen, Norway; 3 British Antarctic Survey, High Cross Madingley Road, Cambridge CB3 0ET, UK

## Abstract

The average rate of melting at the base of the large Filchner-Ronne Ice Shelf in the southern Weddell Sea is currently low, but projected to increase dramatically within the next century. In a model study, melt rates increase as changing ice conditions cause a redirection of a coastal current, bringing warm water of open ocean origin through the Filchner Depression and into the Filchner Ice Shelf cavity. Here we present observations from near Filchner Ice Shelf and from the Filchner Depression, which show that pulses of warm water already arrive as far south as the ice front. This southward heat transport follows the eastern flank of the Filchner Depression and is found to be directly linked to the strength of a wind-driven coastal current. Our observations emphasize the potential sensitivity of Filchner-Ronne Ice Shelf melt rates to changes in wind forcing.

## Abstract (web summary)

Recent modelling studies have indicated that the Filchner-Ronne Ice Shelf in Antarctica might be susceptible to the influx of warmer waters, yet observations evidence is lacking. Here, the authors present observations from the ice shelf, and show that warm water pulses already bathe the shelf.

## Graphical abstract

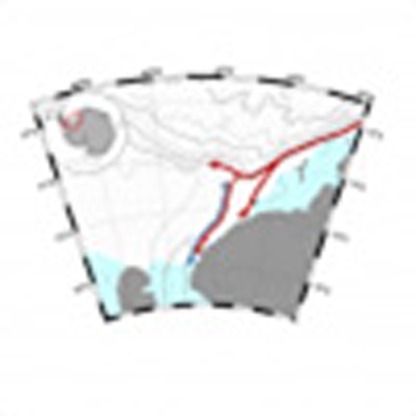

Antarctic ice shelves are thinning at an accelerating rate^1^, leading to reduced buttressing of the ice sheet^2^ with a consequential increase in glacial flow and sea-level rise. The ice shelf thinning is caused by enhanced basal melt^3^ and is therefore linked to increased heat transport from the open ocean towards the ocean cavity beneath the ice shelves. In the areas west of the Antarctic Peninsula where the ice shelves are observed to be rapidly thinning^1^,^3^, warm water of open ocean origin has direct access to the ice-shelf cavities^3^,^4^,^5^. In the southern Weddell Sea, however, where the large Filchner-Ronne Ice Shelf (FRIS) resides (Fig. 1), the water on the wide continental shelf separating the ice-shelf cavity from the deep ocean is cold, and the majority of the water entering the ice-shelf cavity is at the surface freezing point^6^. Basal melt rates in this region are therefore currently low^7^, although recent modelling studies^8^,^9^ suggest that changing ice conditions during the next century will affect the transfer of momentum from the atmosphere to the ocean and redirect the coastal current in a way that will allow the Warm Deep Water (WDW) to access the Filchner Ice Shelf (FIS) cavity through the Filchner Depression (FD, Fig. 1). The melt rates would then increase dramatically—from 0.2 to 4 m per year^8^.

WDW is the Weddell Sea variant of the relatively warm Circumpolar Deep Water that enters the Weddell Sea in the east^10^ and is carried westward in the southern limb of the Weddell gyre. The core of the WDW is typically 0.6–0.9 °C, cooling towards the west^10^,^11^, and found at ∼300 m depth in the interior of the Weddell basin, below a layer that is chilled by the Antarctic atmosphere. Easterly winds along the Antarctic continent cause converging Ekman transport and down-welling along the coast^12^, depressing the WDW and the thermocline to depths below the shelf break. Weaker summer winds allow for a shoaling of the thermocline and a seasonal inflow of WDW along the eastern flank of the FD^13^. FD is a deep trough that cuts across the eastern part of the broad continental shelf in the southern Weddell Sea, and extends beneath FIS to the south (Fig. 1). The depression can act as a direct conduit for warm water southward, similar to the troughs across the continental shelves in the Amundsen and Bellingshausen Sea^14^,^15^. The FD is filled with dense Ice Shelf Water (ISW), that is, water colder than the surface freezing point, which emerges from the ocean cavity beneath FIS^6^. The ISW extends from the sea floor up to above the depth of the FD sill (600 m), confining the warmer, summer inflow to shallower depths. Hydrographic sections from the region in the austral summer typically show a 100 m thick warm layer along the 400 m isobath^6^,^16^; in January 2013 the warm layer was observed as far south as 76S (ref. 17).

The observations presented here show that the warm water eventually reached as far south as the Filchner ice front and that the southward transport of warm water is linked to the strength of a wind-driven coastal current, thus emphasizing the potential sensitivity of FRIS melt rates to changes in wind forcing.

## Results

### Warm water at the Filchner Ice Shelf front

Two oceanographic moorings, M_NORTH_ and M_SOUTH_, ran from January 2013–2014 on the eastern flank of the FD at 77°S (500 m depth) and 78°S (700 m depth), and recorded the arrival of a pulse of warm water (Fig. 2a,c). The warm water is a mixture of WDW and surface water, and is colder than the WDW found off shelf. The warm pulse reaches M_NORTH_ in mid-January, when it was observed at 350 m depth above a 100 m thick layer of ISW. The first, sporadic arrival of warm water at the FIS front (M_SOUTH_) occurs at the beginning of March with a more persistent and deeper presence from the end of the month. On 8th April, the warm layer suddenly deepens to 450 m, followed by a second deepening to 500 m 1 week later. The properties of the warm inflow slowly erode, and from May onwards the temperature at the mooring location is again at or below the surface freezing point. Warm water is present at M_NORTH_ until the beginning of June.

All available temperature and salinity profiles from the front of FIS are shown in Fig. 3, indicating that no WDW has previously been observed at the ice front. In this area, observations from ships are made only during summer (from January to early March), likely too early in the season for warm water to have arrived at the ice front. From February through September 2011, however, a set of oceanographic profiles was collected by a diligent Weddell Seal in close proximity to M_SOUTH_ (ref. 18). The profiles show that during 2011 the warm inflow, while present at the sill^13^, does not reach the front of FIS. Warm water higher in the water column was observed near the ice front in March, 2011 (ref. 18) (and also previously^16^) but this is relatively fresh surface water heated by solar radiation during summer, and it is too shallow to access the ice-shelf cavity.

### The wind-driven coastal current

**Figure 1:**
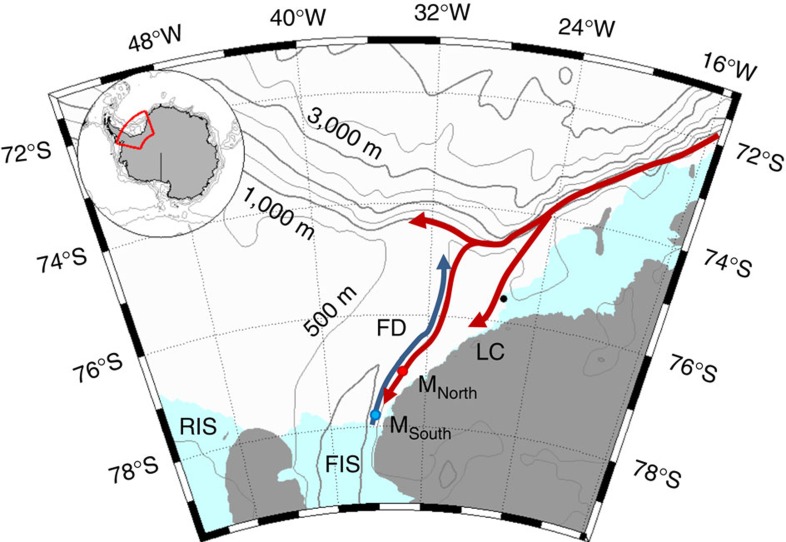
Map. Location map shows the moorings (coloured dots), Halley station (black, 75°35′ S, 26°34′ W), bathymetry^27^ and the circulation in the area^6^,^17^: the blue arrow indicates the flow of cold ISW towards the Filchner sill and the red arrows the path of the coastal/slope front current. The indicated place names are: Filchner Depression (FD), Filchner Ice Shelf (FIS), Luipold coast (LC) and Ronne Ice Shelf (RIS).

The warm inflow through FD that leads to the dramatic increase in basal melt rates in the predictions in ref. 8 is caused by a redirection of the coastal current flowing westward along the southern Weddell Sea coast (Fig. 1). Upstream of the FD, the coastal current is merged with the Antarctic Slope Front current and has a strong barotropic component^19^ that is set up by the prevailing easterly winds (Supplementary Fig. 1a) and the Ekman transport that converges towards the coast. When the continental shelf widens at 27°W the coastal current bifurcates: the inner branch follows the coast^6^ and the outer branch continues along the continental shelf break^20^.

**Figure 2:**
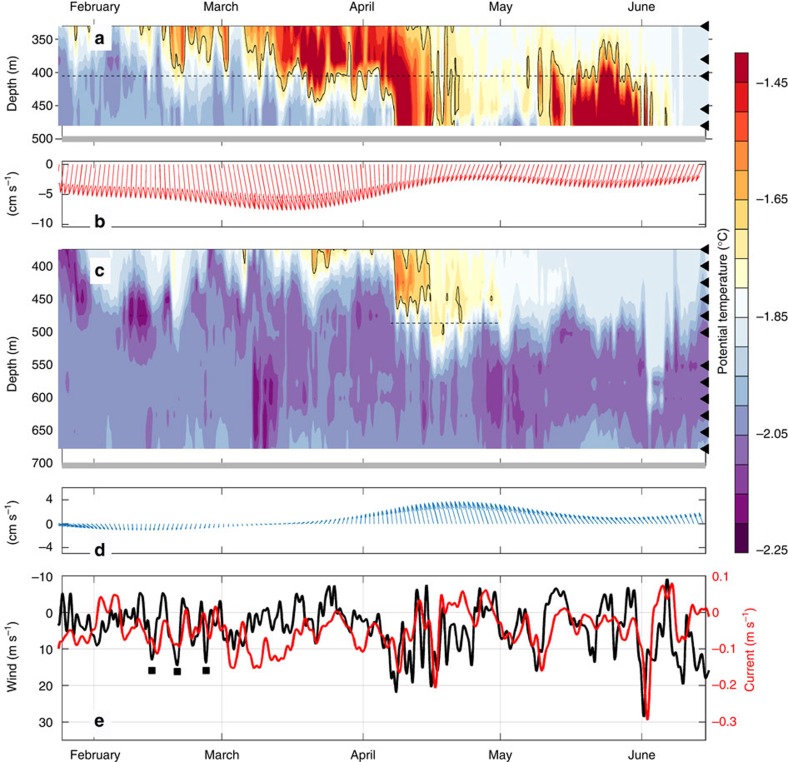
Observations in 2013. Contours of temperature and current vectors (the latter 30 days low-passed) at (**a**–**c**) M_NORTH_ and (**b**–**d**) M_SOUTH_. The sensor depths (temperature) are indicated on the right axis and the −1.7 °C isotherm delineating the Modified Warm Deep Water is highlighted. (**e**) Along slope current at M_NORTH_, and wind observed at Halley along the major wind axis (Methods section). Positive values indicate northward current and southwestward (that is, northwesterly) wind, respectively. Note that the wind axis is reversed. The dashed lines in **a** and **c** mark the depth of the currents measurements displayed in **b**,**d** and **e**, and the period of current observations discussed in the text, the grey lines in **a** and **c** show the sea-floor depth and the black squares in **e** mark the episodes of near gale winds before March.

The currents observed at M_NORTH_ suggest that the current bifurcates a second time: when reaching the divergent isobaths of the FD, part of the current is diverted southward along the eastern flank of the Depression, transporting the warm water present at the shelf break during summer towards the FIS front. The observed southward flow at M_NORTH_ is highly variable and strongly affected by the upstream along-coast wind observed, for example, at the nearby Halley research station (Fig. 2e, Supplementary Figs 1–2). During episodes of strong wind, for example, in April and at the beginning of June (Supplementary Fig. 1b), the southward current at M_NORTH_ exceeds 0.15 m s^−1^, compared with the mean value of 0.03–0.04 m s^−1^. The 16 h-lag (*r*=−0.47, *p*<0.01) between wind and current agrees with the typical lag of 0.5–1.0 days observed in other regions where directly forced wind-driven slope currents are found^21^,^22^.

The effect of strong easterly winds is thus twofold and depends on the time scale considered: on longer (monthly) time scales it depresses the thermocline above the continental slope, shutting off the inflow of warm water towards the continental shelf. On shorter (daily) time scales, it strengthens the coastal current and enhances the southward transport of warm water available on the shelf. The baroclinic response, that is, the depression of the pycnocline, thus depends on the mean wind, while the response of the barotropic current reflects the day to day variability in atmospheric forcing. A combination of generally weak easterly winds, leading to a relaxation of the pycnocline, interrupted by short and intense wind events, setting up strong barotropic currents, would allow warm water to reach the FIS front.

### Warm water in 2011 and 2013

Differences in wind forcing can explain the differences in the observations between 2011 and 2013. The mean wind stress in November–December was weaker (Fig. 4a) in 2013 than in 2011. At the same time, the wind was more variable in 2013 (Fig. 4b, Supplementary Fig. 3 and Supplementary Note 1): there were three episodes of near gale winds at Halley during January–February 2013 with none during the same period in 2011. The weak winds in 2013 potentially allowed for a shallow thermocline and a large warm inflow during early summer that then was advected southward by the wind-driven currents, reaching the FIS front some 350 km to the south about 3 months later. The preconditioning above the slope and the shoaling of the thermocline during spring and early summer is likely the most important factor, allowing for the barotropic current and eddy exchanges^23^ to transport warm water onto the continental shelf.

The inflow dynamics and the thermocline depth are influenced by several factors including the presence of a variable fresh surface layer due to summer sea ice melt^24^, sea ice concentration^8^, shelf salinity^9^,^25^ and interannual variability of the circulation and properties of ISW within the FD. Sea ice conditions in the region differed greatly between the 2 years (Fig. 4c and Supplementary Fig. 4) and, contrary to modelling results which show increased inflow for low shelf salinity^9^, the salinity on the shelf was higher by ∼0.05 in 2013 compared with 2011 (Fig. 4d,e). The relative importance of these factors and the wind stress on the interannual variability of the warm inflow are left as a challenge for the emerging community of high-resolution regional models to disentangle.

### Heat transport and warm water access to the cavity

An upper bound of the southward heat transport is obtained using the mean velocity and the maximum temperature and layer thickness observed at M_NORTH_. Assuming that the inflow width extends from M_NORTH_ to the coast (100 km), the upper bound heat flux is 1.6 TW during the period of inflow (or 0.7 TW when averaged over a year). This is a substantial, geophysically significant flow of heat, enough to melt 70 km^3^ or 65 Gton of ice annually, or about half of the FRIS basal mass budget^26^.

The draft at the ice front is 400–450 m (ref. 27) while the warm water is observed down to 500 m at M_south_. If some of it were to enter the ice-shelf cavity, it would be able to make contact with ∼20% of the FIS base^27^. The mean current at M_SOUTH_ (500 m depth) is however directed northward, away from the ice front, both during the period when warm water is observed at the location and when temperatures are below freezing. The southward flow of warm water presumably lies above shallower isobaths to the east of M_SOUTH_.

The data presented here do not reveal the extent to which the warm water penetrates into the ice-shelf cavity. Relatively strong across ice front tidal currents observed at M_SOUTH_ (0.15 m s^−1^), suggest that the warm water present at the ice front can reach several km into the cavity, enhancing basal melt in the frontal region^28^. Independent evidence suggests that the melt rates in the frontal region can be substantial^29^; basal melt in the outermost 100 km of FRIS accounts for 40% of the total melt below the ice shelf^26^. The warming and shoaling of the warm water core observed since the 1980s (ref. 30) have likely affected the frequency and the heat content of the warm water pulses reaching the FIS. High-resolution time series of ice-shelf thickness show a thinning on the eastern side of the FIS front between 1995 and 2012 (refs 1, 7), while the entirety of the FRIS gained mass during the same period^1^.

The remarkable changes projected to occur in the FRIS cavity during the next century—a 2 °C increase of the water temperature within the ice-shelf cavity and a 20-fold increase in basal melt rates^8^—will have consequences not only for the tributary ice streams and sea-level rise, but for also for the hydrography in a region known to produce a large fraction of Antarctic Bottom Water^31^. We show that the mechanism responsible for the dramatic changes in ref. 8—a redirection of the coastal current—is realistic although important small scale physical processes are not correctly resolved in their coarse model. The wind and ice conditions in 2013 were unusual but not extreme, and it is likely that warm water has occasionally reached the FIS front in other years when observations are not available. Our observations underline the necessity for a continued monitoring of the flow in the FD.

## Methods

### Mooring data

Two bottom-anchored moorings were deployed from January 2013–2014 on the eastern flank of the FD; one at 700 m depth, just north of the FIS front (M_SOUTH_, 77°45.0′ S 36°09.0W) and one about 100 km further north at 500 m depth (M_NORTH_, 77°00.5′ S 34°03.0′ W). The moorings were equipped with Acoustic Doppler Current Profilers (ADCP, Teledyne RD Instruments, 150 kHz and 300 kHz Sentinel), sensors for temperature, conductivity and pressure from Seabird Electronics (SBE37, SBE39 and SBE56) and temperature loggers from Aquatec. At M_SOUTH_, the hydrographic measurements extended from the bottom up to 375 m depth, while the downward-looking ADCP at 475 m gave velocity profiles in the lower part of the water column. The mean horizontal error velocity of the ADCPs where <1 cm s^−1^ at M__NORTH_ and <1.5 cm s^−1^ at M_SOUTH_. Velocity observations where the relative error (|error velocity|/observed speed) is >0.5 are excluded from the analysis. Low abundance of scatterers in the water column during summer (December–February) reduced the data quality below 600 m depth and these data were discarded. At M_NORTH_, hydrographic measurements extend over the deepest 175 m and velocity measurements over the bottom-most 125 m. The coordinate system was rotated clockwise (by 28° and 37° at M_SOUTH_ and M_NORTH,_ respectively) to align the *y* axis with the isobaths locally. Negative *v* is towards the ice-shelf cavity. The time series shown in Fig. 2 are low pass filtered using a 4th order Butterworth filter and a cutoff frequency of 30 days (b,d) or 24 h (e), while the correlation was calculated using a bandpass filtered (24 h—30 days) time series. The significance of the correlation is verified following ref. 32.

### Sea ice

Areal mean sea ice concentration was calculated using data from ref. 33 in two regions; (1) ‘FD': 74–78° S, 25–45° W and (2) ‘Coast': 11–25 W, depths shallower than 3,500 m. Data are available for the period 1978–2014.

### Wind

**Figure 3:**
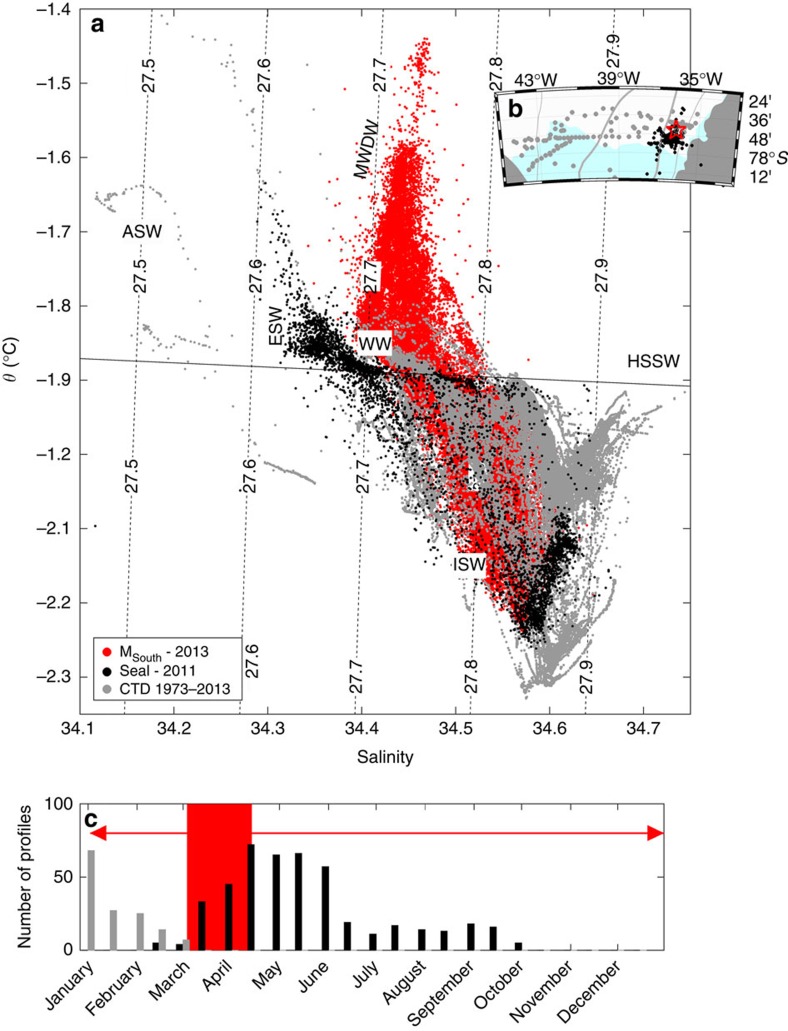
Historical observations from the Filchner ice front. (**a**) Data from M_SOUTH_ 375 m depth in 2013 (red points) and hydrographic profiles from the Filchner ice front collected by a Weddell seal in February to October 2011 (black points) and by ship (south of 77.5S) during summers 1973–2013 (grey points). Only data from depths greater than 200 m are included. The dashed, labelled lines show isopycnals referenced to the surface pressure and the black line shows the surface freezing point. The approximate *θ*/S-properties of the water masses found in the region are indicated: Antarctic surface water (ASW); eastern shelf water (ESW); high salinity shelf water (HSSW); Ice Shelf Water (ISW); Modified Warm Deep Water (MWDW); winter water (WW). The WDW found off the shelf has S≈34.65, *θ*≈0.5 and is off the scale. (**b**) Map showing the location of the M_SOUTH_ (red star) and CTD profiles (coloured dots). (**c**) Histogram showing the temporal distribution of ship (grey) and seal (black) CTD profiles. The red line indicates the duration of the M_SOUTH_ record. The period when modified WDW was observed at M_SOUTH_ is marked in red.

**Figure 4:**
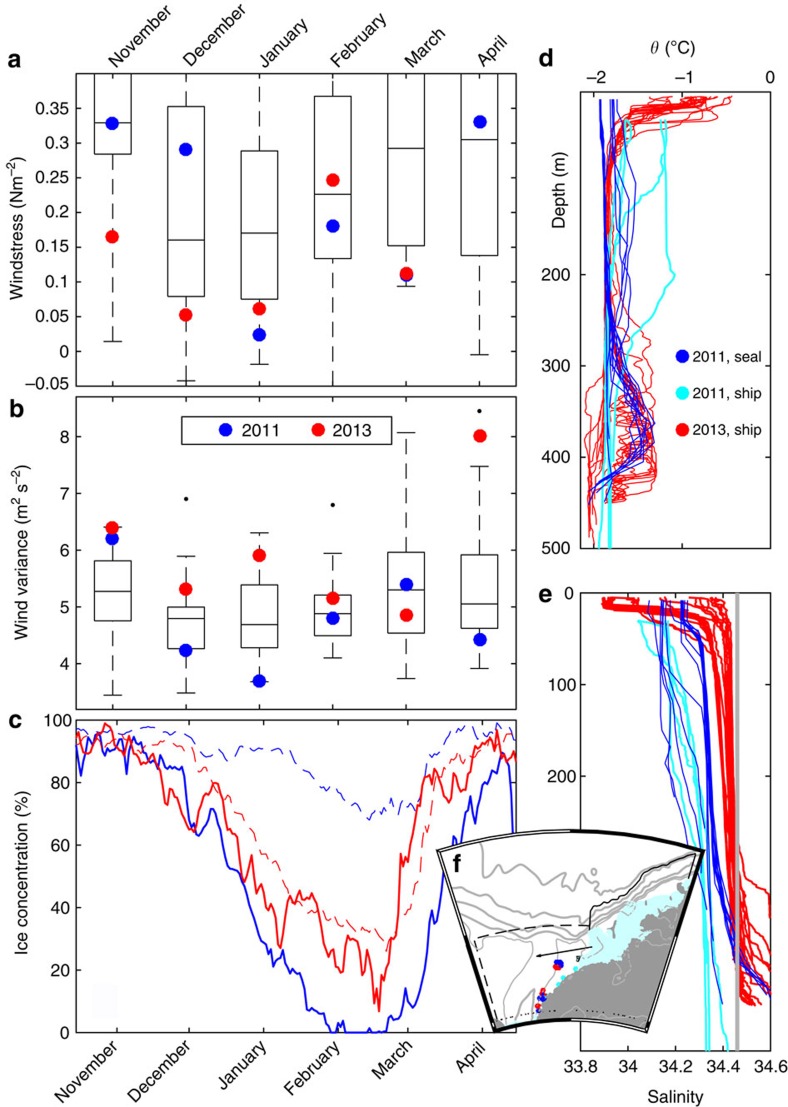
Comparison of year 2011 and 2013. Boxplots of monthly mean (**a**) wind stress and (**b**) variance of wind along the major wind axis from Halley (see **f** and Methods section). Each box shows the 25th and 75th percentile (edges) and the median over 36 years of data (1978–2014). The whiskers show the range of the data, when excluding outliers (black). Values from 2011 are shown in blue and values from 2013 in red. (**c**) Mean sea ice concentration (ref. 33) in the Filchner region (74–78° S, 25–45° W, dashed lines) and from the slope region upstream (11–25° W, shallower than 3,500 m). Boxplots for the ice concentration are shown in Supplementary Fig. 4. Profiles of (**d**) potential temperature and (**e**) salinity obtained on the continental shelf east of the FD in 2011 by seals (blue) and from ship (light blue) and from ship in 2013 (red). Only profiles collected before 31 March each year are included. The thin grey line in **e** shows the salinity at 400 m depth at M_NORTH_ towards the end of the austral winter (August, 2013). (**f**) Map showing the location of the CTD profiles (coloured dots), the boundaries (black lines) for the boxes used when calculating the ice concentration shown in (**c**) the position of Halley (black square) and the major wind axis (black arrow).

Time series of wind stress and variance of the wind were calculated using the observed wind at Halley Station (75°35′ S, 26°34′ W) and the boxplots (Fig. 4a,b) were constructed using monthly mean values from the period 1978 to 2014. The effect varying ice concentration upstream was incorporated in the value of the drag coefficient C_D_ following ref. 34 and the mean ice concentration in ‘Coast' (Fig. 3c). The variance was calculated in 4 week-long sliding windows from the high-pass filtered (3 days) time series. Wind and wind stress were calculated along the major wind axis, shown in Fig. 4, which is rotated 165° counterclockwise from east.

To show that the winds at Halley are representative of a broader region upstream, we have correlated the wind observations from Halley with the along slope wind component from ERA Interim^35^ during the period 1979–2014 (Supplementary Fig. 2c,d). The correlation is high (*r*>0.8) for a large portion of the coastal area upstream of FD.

### Hydrography

CTD (conductivity - temperature - depth) data were collected from RRS *Ernest Shackleton*, 1–10 January 2013, during cruise ES060 (ref. 17), RRS *James Clark Ross*, 19 February–1 March 2011 during cruise JR244 and by sensors attached to Weddell seals in February to October, 2011 (refs 13, 18). References to historical CTD data are given in ref. 17.

### Data availability

The complete mooring records are found in PANGEA (M_SOUTH_, originally called SC at https://doi.pangaea.de/10.1594/PANGAEA.860994 (ref. 36), and M_NORTH_, originally called SD at https://doi.pangaea.de/10.1594/PANGAEA.860995 (ref. 37)). Until the password protection is removed, the part of the data set presented in this publication can be obtained from the corresponding author on request. Sea ice data are available from NOAA/NSIDC Climate Data Record of Passive Microwave Sea Ice Concentration, Version 2. (2013). at http://dx.doi.org/10.7265/N55M63M1 Wind data from Halley Research station are available from the Polar Data Centre, British Antarctic Survey at https://legacy.bas.ac.uk/cgi-bin/metdb-form-1.pl?table_prefix=U_WMC,U_MET&cct=cmet. ERA Interim reanalysis data are available at http://apps.ecmwf.int/datasets/data/interim-full-daily/levtype=sfc/. CTD data from the mooring deployment cruise in 2013 are available in PANGEA, https://doi.pangaea.de/10.1594/PANGAEA.846962 (ref. 38), historical CTD and ‘seal data' are available from the corresponding author on request.

## Additional information

**How to cite this article:** Darelius, E. *et al*. Observed vulnerability of Filchner-Ronne Ice Shelf to wind-driven inflow of warm deep water. *Nat. Commun.* 7:12300 doi: 10.1038/ncomms12300 (2016).

## Supplementary Material

Supplementary Information Supplementary Figures 1-4, Supplementary Note 1 and Supplementary References.
