# Supplementary material for: Observed vulnerability of Filchner-Ronne Ice Shelf to wind-driven inflow of warm deep water
Source: Nat Commun. 2016 Aug 2;7:12300. doi: 10.1038/ncomms12300 (PMC4974661; doi:10.1038/ncomms12300)
Supplement: Supplementary Information — Supplementary Figures 1-4, Supplementary Note 1 and Supplementary References. [file ncomms12300-s1.pdf]

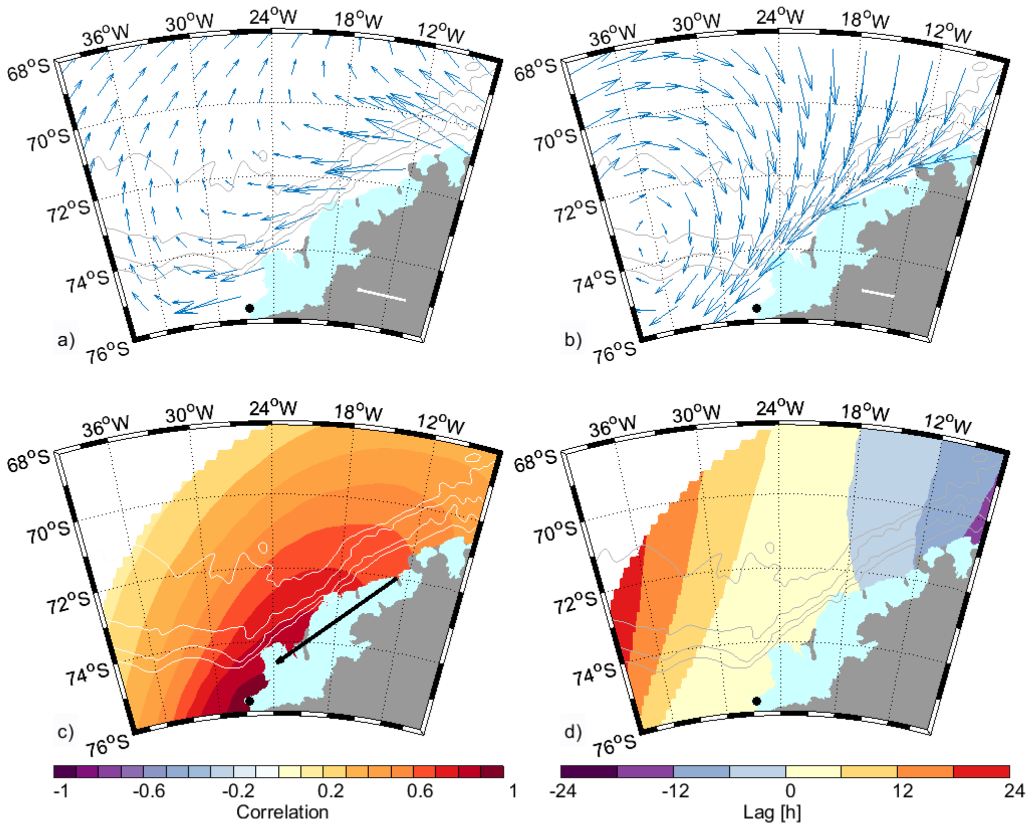

Supplementary Figure 1: Comparison of winds at Halley and along the coast upstream. a) Mean winter wind (June-August, 1979-2014) and b) wind during the storm 1-2 June, 2013 from ERA Interim. The scale vectors in the lower right corners of panel (a) and (b) show 3 and 10 m s^{-1} respectively. c) Maximum lagged correlation between the wind observed at Halley and along slope winds from ERA Interim[1] between 1979-2014. All correlations shown are significant to the 99% level [2] and only values above ocean are shown. d) Lag giving the maximum correlation shown in (c) The black dot shows the location of Halley station and the arrow in (a) shows the direction of the ERA Interim wind component used for the analysis in (c-d).

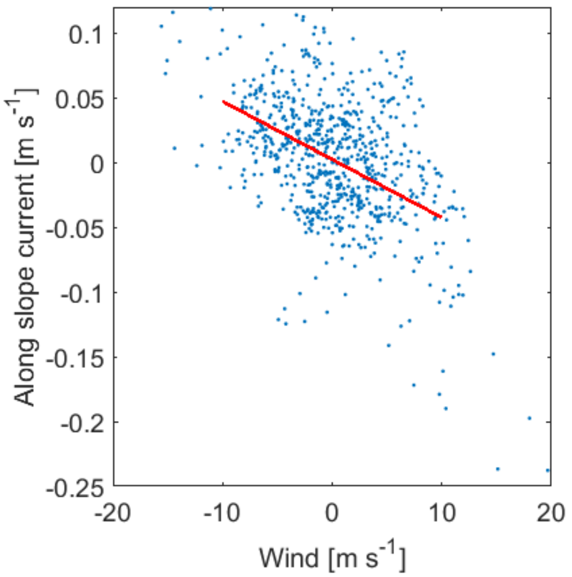

Supplementary Figure 2: Scatter plot of wind and current data. Scatter plot showing the relation between the wind observed between January and June, 2013 at Halley Station and the along slope current observed at M_{NORTH} at 16 h lag. The data are band passed (24 h – 30 days). For clarity only every fifth data point is included. The red line shows the linear regression.

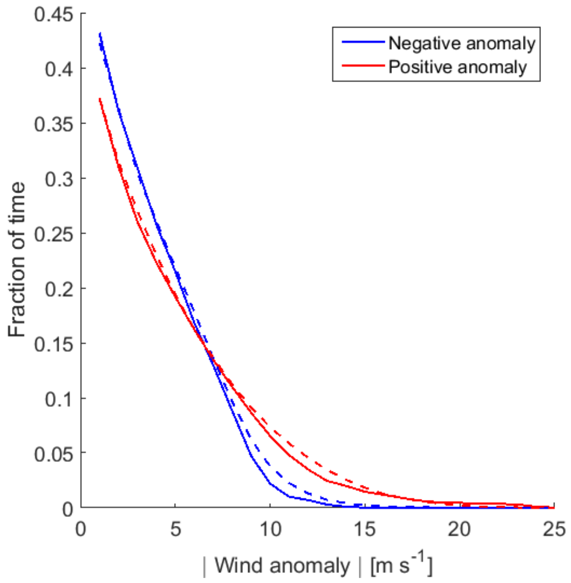

Supplementary Figure 3: Cumulative distribution of wind anomalies. Fraction of time with positive (red) and negative (blue) wind anomalies for which the absolute value is larger than a given value during the period January-June 2013. The dashed lines show the values for the period 1957-2014.

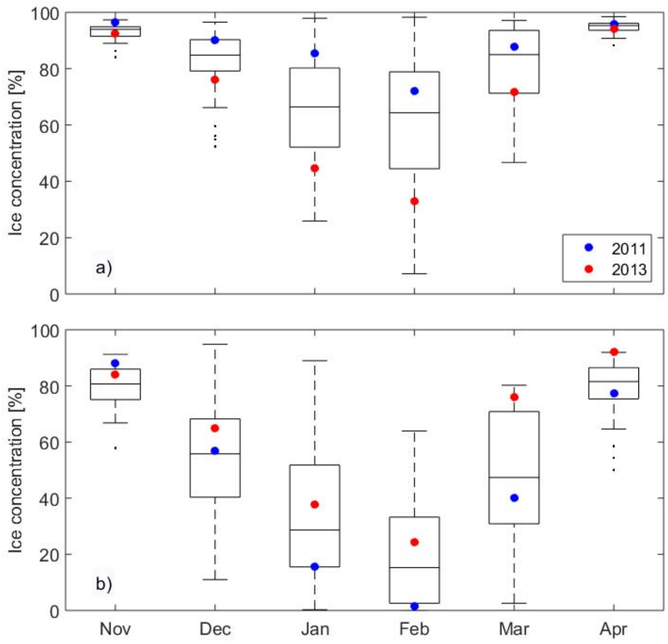

Supplementary Figure 4: Boxplots of monthly mean ice concentration. Monthly ice concentration in a) the Filchner region [74-78S, 25-45W] and from b) the slope region upstream (11-25°W, shallower than 3500 m). Each box shows the 25th and 75th percentile (edges) and the median and includes 36 years of data (1978-2014). The whiskers show the range of the data, when excluding outliers which are shown in black. Values from 2011 are shown in blue and values from 2013 in red.

Supplementary Note 1

The variance of the wind, presented in Fig. 3b, represents deviations from the mean and will lead to acceleration as well as deceleration of the coastal current. The frequency distribution of wind anomalies is however skewed (Supplementary Fig. 3 and Fig 1e), so that strong positive wind anomalies (accelerating the current) are more frequent than strong negative wind anomalies (decelerating the current).

Supplementary References

1. Dee, D. P. *et al.* The ERA-Interim reanalysis: configuration and performance of the data assimilation system. *Q. J. R. Meteorol. Soc.* **137,** 553–597 (2011).

2. Sciremammano, F. J. A Suggestion for the Presentation of Correlations and Their Significance Levels. *J. Phys. Oceanogr.* **9,** 1273–1276 (1979).
